# Food and Nutrition Surveillance System: A time series analysis of data coverage, ultra-processed food consumption and obesity among pregnant adults and adolescents, Brazil, 2008-2022

**DOI:** 10.1590/S2237-96222025v34e20240713.en

**Published:** 2025-11-21

**Authors:** Sthefani da Costa Penha, Antônio Augusto Ferreira Carioca, Maria Soraia Pinto, Lia Silveira Adriano, Mariana Fialho Bastos, Rosiane de Paes Borges Herculano, Bernard Carl Kendall, Ligia Regina Franco Sansigolo Kerr

**Affiliations:** 1 Universidade Federal do Ceará, Faculdade de Medicina, Programa de Pós-graduação em Saúde Pública, Fortaleza, CE, Brazil; 2 Universidade de Fortaleza, Centro de Ciências da Saúde, Programa de Pós-graduação em Saúde Coletiva, Fortaleza, CE, Brazil; 3 Universidade de Fortaleza, Centro de Ciências da Saúde, Curso de Nutrição, Fortaleza, CE, Brazil; 4 Tulane University, Department of Global Community Health and Behavioral Sciences, New Orleans, LA, United States

**Keywords:** Food and Nutritional Surveillance, Maternal Health, Eating, Obesity, Time Series Studies, Vigilancia Alimentaria y Nutricional, Salud Materna, Ingestión de Alimentos, Obesidad, Estudios de Series Temporales

## Abstract

**Objective:**

To assess the temporal trend of coverage and prevalence of ultra-processed food consumption and nutritional status among pregnant women registered on the Food and Nutrition Surveillance System, at the national level and by macro-region.

**Methods:**

This was a time series analysis study, using data on adult and adolescent pregnant women from public production reports and consolidated reports from the system, at the national level and by macro-region, from 2008 to 2022. The annual variation rate of coverage, food consumption and anthropometry indicators was estimated using Prais-Winsten regression, with a 5% significance level.

**Results:**

A total of 319,568 food consumption records and 6,585,600 anthropometric data records were identified. Annual coverage showed an increasing trend of 14.3% for food consumption (95% confidence interval [95%CI] 4.3; 24.7) and 15.2% for anthropometric data (95%CI 10.2; 20.4). Average prevalence of ultra-processed food consumption was 90.0%, remaining stable and high, with greater frequency in the Northeast region. Obesity increased from 13.3% to 29.9% in adult pregnant women and from 4.5% to 10.4% in pregnant adolescents, with an annual variation of 5.2% and 5.9% (p-value <0.001).

**Conclusion:**

There was an increase in the annual coverage of ultra-processed food consumption and anthropometric data of pregnant women registered on the Food and Nutrition Surveillance System. Ultra-processed food consumption prevalence remained stable and high, with a higher frequency of this food group in the Northeast. Percentage obesity increased in the population studied, without, however, showing a direct association between these factors.

## Resumen

**Objetivo:**

Evaluar la tendencia temporal de la cobertura y la prevalencia del consumo de alimentos ultraprocesados, así como el estado nutricional, en mujeres embarazadas registradas en el Sistema de Vigilancia Alimentaria y Nutricional, a nivel nacional y por macrorregión.

**Métodos:**

Se realizó un análisis de series de tiempo, utilizando datos de mujeres embarazadas adultas y adolescentes provenientes de informes públicos de producción e informes consolidados del sistema, a nivel nacional y por macrorregión, de 2008 a 2022. La tasa de variación anual de los indicadores de cobertura, consumo de alimentos y antropometría se estimó mediante la regresión de Prais-Winsten, con un nivel de significancia del 5%.

**Resultados:**

Se identificaron 319.568 registros de consumo de alimentos y 6.585.600 registros de datos antropométricos. La cobertura anual mostró una tendencia creciente del 14,3% para el consumo de alimentos (intervalo de confianza del 95% [IC95%] 4,3; 24,7) y del 15,2% para los datos antropométricos (IC95% 10,2; 20,4). El consumo de alimentos ultraprocesados ​​mostró una prevalencia media del 90,0%, manteniéndose estable y alta, con mayor frecuencia en la región Nordeste. La obesidad aumentó del 13,3% al 29,9% en embarazadas adultas y del 4,5% al ​​10,4% en embarazadas adolescentes, con una variación anual del 5,2% y 5,9% (p-valor <0,001).

**Conclusión:**

Hubo un aumento en la cobertura anual del consumo de alimentos ultraprocesados ​​y de los datos antropométricos de las embarazadas registradas en el Sistema de Vigilancia Alimentaria y Nutricional. La prevalencia del consumo de alimentos ultraprocesados ​​se mantuvo estable y alta, con una mayor frecuencia de este grupo de alimentos en el Nordeste. El porcentaje de obesidad aumentó en la población estudiada, sin que, sin embargo, se observara una asociación directa entre estos factores.

**Ethical aspects**

This research respected ethical principles, having obtained the following approval data:

Research Ethics Committee: Universidade de Fortaleza

Opinion number: 4348452

Approval date: 16/4/2020

Certificate of Submission for Ethical Appraisal: 31540320.9.1001.5052

Informed Consent Form: Not applicable.

## Introduction

Pregnancy produces physiological changes to adapt the woman’s body to its needs and fetal development (1). In this scenario, ultra-processed food consumption can result in reduced nutritional quality, as well as the development of obesity, gestational diabetes and pre-eclampsia in pregnant women, in addition to low birth weight and changes in neurodevelopment in their babies (2-5). Ultra-processed food consumption is influenced by sociodemographic factors, including younger maternal age, low education levels and multiparity (6).

Food and Nutrition Surveillance, the third guideline of the National Food and Nutrition Policy, is essential because it enables monitoring of food and nutrition and their determinants through the Food and Nutrition Surveillance System (7). This system was launched in 2008 and records and disseminates information on food consumption and the nutritional status of the population monitored in Primary Care services of the Brazilian National Health System (8).

Meals should prioritize inclusion of unprocessed or minimally processed foods, with daily intake of fruit, vegetables, beans and legumes, restricting ultra-processed foods and beverages with added sugar as part of the diet (9).

In the usual diet of Brazilian pregnant women, 21.0% of food consumed is ultra-processed (10). It can be seen that, as the share of these foods increases, there is a reduction in the intake of unprocessed or minimally processed foods. The increased share of ultra-processed food is associated with a higher intake of calories, simple carbohydrates, sodium and fats. At the same time, there is a decrease in consumption of proteins, complex carbohydrates, iron, folate, selenium, zinc and potassium, thus compromising the quality of food during this period (11).

High prevalence of overweight was found among pregnant adolescents in Brazil as a whole and also in the country’s macro-regions between 2008 and 2018. As such, there is evidence of changes in the nutrition profile over the years, associated with an increase in complications during pregnancy and birth (12,13).

The increasing prevalence of diseases related to excessive consumption of ultra-processed food and inadequate nutritional status, especially among pregnant women, highlights the need for continuous monitoring of food intake and nutritional status. In this sense, the aim is to guide public policies focused on food and nutrition and improve nutritional interventions, contributing to the prevention of adverse conditions among mothers and their children.

This study aimed to evaluate the temporal trend in coverage and prevalence of ultra-processed food consumption and nutritional status and the correlation between intake of ultra-processed food and obesity in adult and adolescent pregnant women registered on the Food and Nutrition Surveillance System, at the national level and by macro-region.

## Methods

### Study design

This was a time series analysis study using secondary data obtained via the Food and Nutrition Surveillance System platform (web version), for the period 2008-2022. The units of analysis included Brazil as a whole, the country’s five macro-regions (North, Northeast, Midwest, Southeast and South), its 26 states and its Federal District. Data were collected from the system on April 23, 2023.

### Setting

The Food and Nutrition Surveillance System was first made available by the Ministry of Health in 2004. In 2008, a version for online access was released with the aim of improving the system, including the recording and dissemination of information on food intake and nutritional assessment of the population monitored in Primary Health Care. In 2017, considering its significant role, version 3.0 of the system was released, aiming to improve the integration of the system with the e-SUS Primary Care system (8).

The website compiles information about food and nutrition surveillance actions, obtained from anthropometric indexes and food intake indicators collected and recorded by health professionals, the Bolsa Família Conditional Cash Transfer Program Management System, the Food and Nutrition Surveillance System (web version), and the e-SUS Primary Care system (14). Public access can be made using filters, including month and year, region, and stage of life. Based on this, data is then generated on an electronic spreadsheet.

### Participants

Data on coverage of registered adult and adolescent pregnant women held on the Food and Nutrition Surveillance System were analyzed. When conducting this study, we took into consideration separately the stage of life of each pregnant woman, given that the system classifies them together simply as pregnant women (15).

### Variables

The total coverage rate of pregnant women was calculated by dividing the number of food consumption records from 2015 to 2022 and nutritional status records from 2008 to 2022 held on the Food and Nutrition Surveillance System (web version) by the number of live births in that period, multiplied by 100, based on a previous study (16). In order to calculate the estimate of pregnant women, we took the number of live births held on the Live Birth Information System, applying filters, including year and region of Brazil.

Food intake was assessed using food consumption indicators. These were analyzed using forms to obtain information about the food consumed the previous day, which was collected by professionals who make up Primary Health Care teams. Three forms were made available, one for children under 6 months, one for children aged 6-23 months and another for children aged 2 years or older, adolescents, pregnant women, adults and the elderly. In this study, we used the form for pregnant women. The ultra-processed food consumption indicator corresponded to at least one marker of unhealthy food consumption on the previous day, with the following options: sugar-sweetened beverages; instant noodles, packaged snacks or savory biscuits; hamburgers and/or sausages; and stuffed cookies, sweets or treats (17).

Nutritional status was measured by body mass index (BMI) by gestational week and assessed according to the classification proposed by Atalah, a parameter adopted by the Food and Nutrition Surveillance System in Brazil and recognized in technical publications of the Ministry of Health. This classification made it possible to assess nutritional evolution during pregnancy (18) and included:

- I – low weight (BMI lower than or equal to the values shown in the low weight nutritional status column);
- II – adequate weight (BMI between the values shown in the low weight and adequate weight nutritional status column); and
- III – overweight or obesity (BMI between the values shown in the overweight or obesity nutritional status column).

The numerical cutoff points for nutritional status classification varied according to gestational week, making it impossible to adopt fixed values ​​for each category. This index was the result of body weight in kilograms divided by squared height in meters.

The gestational week was determined by taking the date of the last menstrual period, this being information that is present and mandatory in the questionnaire. This information is usually known by the expectant mother, but it can also be collected through tests performed during pregnancy, or, in the absence of reliable information, the date should be estimated based on the last month of menstruation (15).

### Data source

Coverage data were assessed through public production reports. Information on food intake and nutritional status was analyzed by means of reports consolidated by year obtained via the Food and Nutrition Surveillance System (web version) website (https://sisaps.saude.gov.br/sisvan/relatoriopublico/index), whereby the analysis codes were defined according to the data in question (food consumption or nutritional status), reference year, reference month, region, age group and stage of life.

### Bias

In order to minimize the risk of bias, the Food and Nutrition Surveillance System protocols were applied to exclude implausible values. In addition, data standardization protocols exist.

### Study size

We analyzed data aggregated by the country’s macro-regions and for Brazil as a whole, with 6,585,600 records for anthropometry and 319,568 records for food consumption of pregnant women. Percentage coverage of the Food and Nutrition Surveillance System was calculated by the annual number of records divided by the number of live births in that period, multiplied by 100. This population was estimated based on the number of live births, collected from the Live Birth Information System, for each year, between 2008 and 2022.

### Statistical methods

The data collected from the Food and Nutrition Surveillance System web platform for each macro-region and reference year were expressed as relative values ​​(%). Coverage and prevalence of ultra-processed food consumption and obesity (dependent variable) were calculated according to macro-region and for Brazil as a whole for the reference year (independent variables).

In order to determine the temporal variation of the indicators, the prevalence of each stratum was calculated by year. Then, Prais-Winsten regression models were used, generating a beta coefficient (β) and its 95% confidence intervals, assuming a 5% significance level. This method is recommended for ecological studies in order to minimize self-correction of regression residuals between the years of analysis (19). To calculate average annual variation in the coverage of ultra-processed food consumption and obesity, we used the annual growth rate (AGR) formula: 

AGR=[-1 + (10^β^)] x 100,

where β is the result of Prais-Winsten regression and base-10 logarithm. A stable trend was indicated based on a non-significant p-value (p-value≥0.05) and an increasing or decreasing trend when the p-value was significant (p-value<0.05), according to positive or negative annual variation. This information for each of Brazil’s federative units was used for Pearson’s correlation between the annual growth rates of ultra-processed food intake and obesity between 2015 and 2022. We used Stata version 11.2 (Stata Corp, College Station, Texas, United States) to perform all the statistical analyses.

## Results

Between 2008 and 2022, 6,585,600 anthropometric records and 319,568 food consumption records of pregnant women registered on the Food and Nutrition Surveillance System were identified. From 2015 to 2022, there was an increase in the coverage of data for ultra-processed food consumption indicators from 0.7% to 2.3% (AGR 14.3%; 95%CI 4.3; 24.7). Of the total records of data for food consumption indicators, 49.4% related to the Southeast, 19.5% related to the Northeast, and 12.3% related to the South. Anthropometric data coverage increased from 3.9% in 2008 to 34.1% in 2022, with a significant annual variation of 15.2% (95%CI 10.2; 20.4) (Table 1). Most anthropometric data records were found for the Northeast (35.0%), followed by the Southeast (32.3%) and Northern (11.6%) regions.

**Table 1:** Annual growth rate (AGR) and 95% confidence interval (95%CI) of the coverage of the ultra-processed food consumption indicator (%) and anthropometric data (%) for pregnant women registered on the Food and Nutrition Surveillance System. Brazil, 2015-2022 (n=8,872,011)

Year	n	Ultra-processed food consumption (n)	Ultra-processed food consumption (%)	Coverage of the ultra-processed food consumption indicator (%)
2015	21,163	16,753	82.0	0.7
2016	32,308	31,137	81.0	1.1
2017	42,120	33,144	81.0	1.4
2018	42,993	32,270	76.0	1.5
2019	38,561	29,243	77.0	1.4
2020	33,561	25,059	76.0	1.2
2021	49,046	39,506	82.0	1.9
2022	59,816	45,382	77.0	2.3
AGR (95%CI)				14.1 (4.3; 24.7)
	n	Obesity (n)	Obesity (%)	Coverage of the anthropometric data (%)
2008	115,406	115,406	11.2	3.9
2009	209,099	209,099	12.6	7.3
2010	252,620	252,620	13.1	8.8
2011	318,150	318,150	14.4	10.9
2012	405,533	405,533	15.2	14.0
2013	483,965	483,965	15.5	16.7
2014	501,638	501,638	16.3	16.8
2015	692,975	692,975	16.9	23.0
2016	848,533	848,533	17.2	29.7
2017	733,089	733,089	18.2	25.1
2018	728,695	728,695	19.6	24.7
2019	629,527	629,506	20.4	22.1
2020	1,031,416	1,031,380	23.1	37.8
2021	1,047,717	1,047,711	23.9	39.1
2022	873,648	873,586	24.4	34.1
AGR (95%CI)				15.2 (10.2; 20.4)

Annual percentage variation in ultra-processed food consumption remained stable. However, these foods accounted for an average of 90.0% of the diet of pregnant Brazilian women. In the South, the percentage of registered pregnant adults who consumed hamburgers and/or sausages increased by an average of 7.9% per year (p-value 0.005), with a 3.6% increase per year in the consumption of instant noodles, packaged snacks or savory biscuits (p-value 0.006) and a 3.3% increase in the consumption of stuffed cookies, sweets or treats (p-value 0.003). Consumption of sugar-sweetened beverages increased by 1.5% per year in the Northeast, whereas in the North it reduced by 1.9% (p-value 0.020) (Tables 2 and 3).

In the Northeast, the percentage of adult pregnant women who consumed instant noodles, packaged snacks or savory biscuits on the previous day increased, on average, by 1.8% per year (p-value<0.001). Similarly, there was an increase of 1.6% per year in consumption of stuffed cookies, sweets or treats (p-value 0.002). In the North, there was a 3.8% decrease per year in the consumption of instant noodles, packaged snacks or savory biscuits (p-value 0.037) and a 2.6% decrease in the consumption of stuffed cookies, sweets or treats (p-value 0.034) (Table 2).

**Table 2:** Annual growth rate (AGR) and p-value of the ultra-processed food consumption indicator (%) among adult pregnant women registered on the Food and Nutrition Surveillance System. Brazil and its macro-regions, 2015-2022 (n=319,715)

Year	Midwest (%)	Northeast (%)	North (%)	Southeast (%)	South (%)	Brazil (%)
2015	85.0	72.0	80.0	82.0	84.0	81.0
2016	82.0	70.0	77.0	82.0	83.0	80.0
2017	81.0	72.0	75.0	82.0	82.0	79.0
2018	76.0	71.0	78.0	74.0	82.0	75.0
2019	79.0	73.0	73.0	76.0	83.0	76.0
2020	81.0	71.0	74.0	76.0	79.0	75.0
2021	87.0	76.0	77.0	82.0	88.0	81.0
2022	95.0	92.0	90.0	92.0	93.0	92.0
AGR (p-value)	1.5 (0.324)	2.7 (0.074)	1.0 (0.426)	1.2 (0.443)	1.2 (0.214)	1.5 (0.345)
**Consumption of hamburgers and/or sausages**						
2015	43.0	33.0	44.0	35.0	36.0	36.0
2016	33.0	32.0	35.0	32.0	37.0	33.0
2017	34.0	33.0	31.0	32.0	37.0	33.0
2018	32.0	33.0	34.0	29.0	35.0	31.0
2019	37.0	32.0	26.0	36.0	43.0	35.0
2020	47.0	34.0	26.0	37.0	44.0	36.0
2021	61.0	46.0	39.0	49.0	66.0	50.0
2022	41.0	37.0	34.0	41.0	51.0	41.0
AGR (p-value)	4.5 (0.210)	3.5 (0.040)	-2.5 (0.436)	4.7 (0.057)	7.9 (0.005)	4.1 (0.078)
**Consumption of sugar-sweetened beverages**						
2015	65.0	49.0	54.0	64.0	65.0	61.0
2016	63.0	47.0	59.0	62.0	62.0	60.0
2017	60.0	46.0	54.0	62.0	60.0	58.0
2018	58.0	47.0	57.0	57.0	60.0	55.0
2019	57.0	50.0	53.0	58.0	63.0	56.0
2020	61.0	49.0	51.0	57.0	59.0	55.0
2021	72.0	54.0	55.0	63.0	70.0	62.0
2022	61.0	50.0	52.0	60.0	64.0	57.0
AGR (p-value)	0.2 (0.874)	1.5 (0.034)	-1.9 (0.020)	-0.7 (0.371)	0.7 (0.345)	-0.6 (0.499)
**Consumption of instant noodles, packaged snacks or savory biscuits**						
2015	43.0	33.0	44.0	35.0	36.0	36.0
2016	33.0	32.0	35.0	32.0	37.0	33.0
2017	34.0	33.0	31.0	32.0	37.0	33.0
2018	32.0	33.0	34.0	29.0	35.0	31.0
2019	34.0	35.0	30.0	30.0	40.0	33.0
2020	38.0	35.0	30.0	32.0	39.0	34.0
2021	42.0	36.0	31.0	34.0	46.0	36.0
2022	36.0	36.0	31.0	34.0	45.0	36.0
AGR (p-value)	0.2 (0.905)	1.8 (<0.001)	-3.8 (0.037)	-0.1 (0.938)	3.6 (0.006)	0.5 (0.677)
**Consumption of stuffed cookies, sweets or treats**						
2015	49.0	32.0	43.0	45.0	42.0	43.0
2016	42.0	34.0	40.0	44.0	43.0	42.0
2017	43.0	37.0	39.0	45.0	43.0	43.0
2018	40.0	36.0	38.0	39.0	42.0	39.0
2019	40.0	37.0	35.0	40.0	47.0	40.0
2020	39.0	35.0	33.0	40.0	43.0	38.0
2021	53.0	39.0	37.0	44.0	55.0	44.0
2022	42.0	36.0	36.0	42.0	50.0	41.0
AGR (p-value)	0.0 (0.992)	1.6 (0.026)	-2.6 (0.034)	-1.0 (0.353)	3.3 (0.003)	-0.5 (0.517)

In the pregnant adolescent group, percentage variation in consumption of sugar-sweetened beverages on the previous day decreased by 1.0% between 2015 and 2022 in Brazil as a whole (p-value 0.034) and by 1.6% in the North (p-value 0.027). However, in those seven years, the number of pregnant adolescents who consumed hamburgers and/or sausages grew by 4.6% per year in the Northeast (p-value 0.005). In the South, there was a 3.5% increase per year in consumption of hamburgers and/or sausages (p-value 0.003) and a 1.5% increase in consumption of stuffed cookies, sweets or treats (p-value 0.002) (Table 3). 

**Table 3:** Annual growth rate (AGR) and p-value of the ultra-processed food consumption indicator (%) among pregnant adolescents registered on the Food and Nutrition Surveillance System. Brazil and its macro-regions, 2015-2022 (n=42,911)

Year	Midwest (%)	Northeast (%)	North (%)	Southeast (%)	South (%)	Brazil (%)
2015	90.0	80.0	84.0	90.0	93.0	87.0
2016	88.0	82.0	83.0	89.0	88.0	87.0
2017	85.0	83.0	79.0	88.0	90.0	86.0
2018	80.0	77.0	83.0	83.0	89.0	82.0
2019	88.0	79.0	77.0	83.0	88.0	81.0
2020	87.0	79.0	79.0	84.0	86.0	82.0
2021	90.0	81.0	83.0	87.0	90.0	85.0
2022	95.0	90.0	89.0	91.0	91.0	91.0
AGR (p-value)	0.7 (0.415)	0.9 (0.293)	0.5 (0.596)	0.0 (0.959)	-0.2 (0.568)	0.5 (0.633)
**Consumption of hamburgers and/or sausages**						
2015	47.0	32.0	38.0	41.0	46.0	40.0
2016	39.0	37.0	31.0	42.0	43.0	39.0
2017	39.0	37.0	40.0	41.0	47.0	39.0
2018	35.0	34.0	33.0	39.0	50.0	38.0
2019	33.0	39.0	30.0	41.0	50.0	39.0
2020	51.0	44.0	30.0	45.0	46.0	42.0
2021	63.0	47.0	42.0	49.0	58.0	48.0
2022	49.0	42.0	38.0	44.0	56.0	44.0
AGR (p-value)	3.3 (0.409)	4.6 (0.005)	0.7 (0.733)	1.8 (0.130)	3.5 (0.003)	2.2 (0.083)
**Consumption of sugar-sweetened beverages**						
2015	69.0	53.0	61.0	71.0	75.0	67.0
2016	67.0	58.0	64.0	70.0	69.0	67.0
2017	69.0	57.0	59.0	70.0	71.0	66.0
2018	62.0	53.0	64.0	65.0	69.0	63.0
2019	66.0	56.0	57.0	66.0	71.0	63.0
2020	71.0	55.0	54.0	67.0	68.0	62.0
2021	70.0	57.0	59.0	67.0	69.0	64.0
2022	74.0	56.0	57.0	67.0	71.0	63.0
AGR (p-value)	1.0 (0.256)	0.2 (0.673)	-1.6 (0.027)	-0.9 (0.094)	-0.5 (0.115)	-1.0 (0.034)
**Consumption of instant noodles, packaged snacks or savory biscuits**						
2015	48.0	43.0	49.0	47.0	50.0	47.0
2016	43.0	44.0	41.0	43.0	47.0	43.0
2017	45.0	42.0	38.0	41.0	47.0	42.0
2018	39.0	39.0	43.0	38.0	47.0	40.0
2019	44.0	42.0	37.0	38.0	51.0	40.0
2020	45.0	43.0	36.0	41.0	48.0	41.0
2021	51.0	43.0	41.0	43.0	49.0	44.0
2022	51.0	42.0	43.0	41.0	53.0	43.0
AGR (p-value)	1.6 (0.325)	-0.2 (0.788)	-1.4 (0.382)	-1.6 (0.291)	0.9 (0.117)	-1.0 (0.404)
**Consumption of stuffed cookies, sweets or treats**						
2015	58.0	46.0	50.0	57.0	55.0	54.0
2016	52.0	49.0	50.0	56.0	54.0	53.0
2017	53.0	50.0	46.0	55.0	58.0	53.0
2018	48.0	45.0	45.0	51.0	55.0	50.0
2019	48.0	47.0	43.0	48.0	59.0	48.0
2020	54.0	46.0	41.0	51.0	54.0	49.0
2021	60.0	48.0	46.0	54.0	63.0	52.0
2022	56.0	45.0	45.0	51.0	59.0	50.0
AGR (p-value)	0.3 (0.869)	-0.6 (0.209)	-1.7 (0.130)	-1.5 (0.152)	1.5 (0.002)	-1.1 (0.149)

Between 2008 and 2022, prevalence of obesity in adult pregnant women increased from 13.3% to 29.9%, with an increase of 5.2% per year in all macro-regions and in Brazil as a whole (p-value<0.001) (Table 4). In the pregnant adolescent group, there was a 5.9% annual increase in obesity, increasing from 4.5% in 2008 to 10.4% in 2022 (p-value<0.001) (Table 5).

**Table 4:** Annual growth rate (AGR) and p-value of the prevalence (%) of obesity among adult pregnant women registered on the Food and Nutrition Surveillance System. Brazil and its macro-regions, 2008-2022 (n=1,566,144)

Year	Midwest (%)	Northeast (%)	North (%)	Southeast (%)	South (%)	Brazil (%)
2008	14.1	9.8	9.4	15.6	17.1	13.3
2009	14.5	11.0	8.9	16.7	18.3	14.9
2010	15.2	11.5	8.9	18.1	18.4	15.5
2011	17.4	13.1	10.6	19.6	19.8	16.9
2012	18.1	14.9	11.2	20.1	21.6	17.7
2013	19.0	14.5	11.9	20.0	22.2	18.1
2014	20.2	15.7	13.2	20.7	22.9	19.0
2015	23.1	16.8	14.7	21.8	24.0	19.6
2016	23.8	17.6	15.0	22.8	24.5	20.2
2017	22.2	18.7	15.9	24.3	26.1	21.3
2018	23.6	20.4	17.3	25.6	27.6	22.5
2019	24.4	21.2	18.2	26.1	27.8	23.2
2020	25.5	23.6	20.5	29.4	29.3	26.1
2021	25.7	24.2	21.0	29.6	29.7	26.6
2022	26.0	24.4	21.6	29.8	29.8	29.9
AGR (p-value)	4.6 (<0.001)	6.8 (<0.001)	7.1 (<0.001)	4.7 (<0.001)	4.0 (<0.001)	5.2 (<0.001)

**Table 5:** Annual growth rate (AGR) and p-value of the prevalence (%) of obesity among pregnant adolescents registered on the Food and Nutrition Surveillance System. Brazil and its macro-regions, 2008-2022 (n=139,144)

Year	Midwest (%)	Northeast (%)	North (%)	Southeast (%)	South (%)	Brazil (%)
2008	4.3	3.4	2.8	4.6	6.4	4.5
2009	5.2	3.0	2.2	5.5	7.1	5.0
2010	4.8	3.6	2.4	6.1	6.9	5.2
2011	6.3	4.1	2.8	7.2	7.9	6.0
2012	6.7	4.5	3.0	6.9	8.5	6.2
2013	6.8	4.5	3.3	7.0	8.9	6.3
2014	6.7	4.9	3.6	7.4	9.3	6.6
2015	10.5	5.9	4.8	8.0	9.9	7.1
2016	9.9	6.2	4.7	8.0	10.0	7.1
2017	7.8	6.4	4.8	8.8	10.4	7.3
2018	8.1	7.2	5.3	9.6	11.2	7.9
2019	8.5	7.7	6.0	10.2	11.6	8.5
2020	9.5	8.8	6.9	11.5	13.2	9.7
2021	10.3	9.3	7.2	12.4	14.3	10.4
2022	10.6	9.4	6.9	12.1	13.9	10.4
AGR (p-value)	5.9 (<0.001)	8.6 (<0.001)	9.1 (<0.001)	6.7 (<0.001)	5.7 (<0.001)	5.9 (<0.001)

The annual increase rates for the federative units regarding consumption of ultra-processed food and obesity in adult pregnant women did not present a significant correlation (r=0.19; p-value 0.382). Concomitantly, no significant correlation was observed between these variables in pregnant adolescents (r=

-0.07; p-value 0.763).

## Discussion

This study found an increase in the coverage of food consumption indicators, with most records referring to the Southeast region, and an increase in the coverage of anthropometric data, with the highest frequency of data for the Northeast region. Intake of ultra-processed food by pregnant women in Brazil remained stable, but a higher frequency of this food group was seen in the Northeast, with greater inclusion of hamburgers and/or sausages in the diet of adult pregnant women in the South, while there was a decrease in consumption of sugar-sweetened beverages by pregnant women in Brazil as a whole and in the North. Between 2008 and 2022, there was an increase in the prevalence of obesity in the population studied, which may reflect the continued intake of ultra-processed food in Brazil, these being foods that have excess calories and low nutritional quality. These findings reinforce the importance of Food and Nutrition Surveillance as a fundamental strategy for monitoring the determinants of maternal and child health and guiding actions within the health system.

The results presented have limitations that should be considered when interpreting them. Since this is a time series analysis study, it is not possible to extrapolate its aggregated conclusions to the individual level. Use of secondary data is inherently incomplete and inconsistent. However, the Food and Nutrition Surveillance System is a significant source of data for conducting food and nutrition surveillance, as it has been improved as a tool for coverage and information of guaranteed quality, becoming essential for management and the creation of public policies. This study used forms that assessed food consumption on the previous day through objective questions, thus minimizing memory biases.

The increased coverage of the indicators of consumption of ultra-processed food and anthropometric data among the pregnant women analyzed suggested better nutritional monitoring. Despite the increasing trend of coverage, these percentages were below expected regarding their representativeness of this population at the national level. However, the literature indicated an evolution in coverage after the inclusion of e-SUS Primary Care data on the system (8,13). 

Among users registered on the Food and Nutrition Surveillance System in 2008 and 2017, there was a greater annual variation in the increase in coverage of the group of pregnant women (19). These results can be explained by the greater monitoring of data collected from this population during prenatal care by the Family Health Strategy. Assessment of nutritional status is one of the conditions regarding health required in order to have access to the Bolsa Família Program (13), which provides income transfer in order to alleviate social inequalities.

Most records of food consumption indicators came from the Southeast, while anthropometric data were more frequently recorded in the Northeast. This pattern was also identified in an ecological study on the coverage of the Food and Nutrition Surveillance System and the nutritional status of the elderly population in Brazil, which showed the greatest variations in data availability in these two regions (20). These results may be related to socioeconomic differences between these regions: the Southeast, because it has greater infrastructure and resources, can better absorb the progress made with the system; and the Northeast, given that it concentrates a greater number of Bolsa Família Program beneficiaries, may prioritize collection of anthropometric data for monitoring the nutritional status of the population.

Although stability of ultra-processed food consumption by pregnant women in Brazil was found, the 90.0% prevalence rate indicated high intake of this food group, above the Brazilian population’s average consumption of 18.0% (21). When comparing Brazilian regions from 2015 to 2022, the highest frequency of ultra-processed food consumption was identified in the Northeast. The highest share of hamburger and/or sausage consumption was found in the South and Northeast regions, while the lowest intake of instant noodles, packaged snacks or savory biscuits by adult pregnant women was found in the North. Consistent with these results, a study found that Italian women with twin pregnancies presented excessive consumption of processed meat and fish (22). High consumption of ultra-processed food and this continuing over time are characteristic of the hegemonic food system, in which there is dominance in the structure of production, distribution and consumption of food worldwide as a result of industrialization and globalization (23).

In addition, the COVID-19 pandemic changed eating habits, encouraging greater inclusion of ultra-processed foods to the detriment of unprocessed and minimally processed foods, as evidenced by the increase in percentages in 2020, the year in which the pandemic period began. At that time, an increase in food storage was observed among adults with obesity, which was seen to be a result of the economic difficulties and social isolation experienced. In this category, foods with higher energy density, lower nutritional quality and longer shelf life stood out, such as ultra-processed foods (24).

The reduction in the consumption of sugar-sweetened beverages by pregnant women in Brazil overall and in the Northern region coincides with that found by the 2017-2018 Household Budget Survey (25), as well as the data provided by the 2013 and 2023 Chronic Disease Risk and Protective Factors Surveillance Telephone Surveys (21,26), which found a decrease in the share of soft drinks in Brazilian households in recent years. It is suggested that the planning and improvement of public policies, such as the publication of the second edition of the Dietary Guidelines for the Brazilian Population in 2014 and its respective booklets, are essential for these changes.

An increasing trend of obesity was observed in adult and adolescent pregnant women in Brazil overall and across all macro-regions, reinforcing the findings of the ecological study with pregnant adolescents participating in the Bolsa Família Program, which found an increase in prevalence of overweight and obesity between 2008 and 2018, with an annual variation of 8.0% (12). The increase in prevalence of obesity among pregnant women in Brazil is characteristic of the so-called nutrition transition, already observed in the adult population and intensified by the COVID-19 pandemic (27). The evolution of gestational weight gain is influenced by nutritional status prior to pregnancy, as was observed in the weight evolution of Portuguese pregnant women (28).

Socioeconomic conditions, such as education and income, are identified as the main factors that exert progressive effects of the pre-gestational environment on weight gain during pregnancy (29). In addition to this, there is the current scenario of a global syndemic, which is defined as a set of pandemics that affect the population in the same place and period, including obesity, malnutrition, climate change and food and nutrition insecurity (characterized by the consumption of high-calorie foods that are lacking in essential nutrients) (30).

In this context, the importance of improving access to adequate care in Primary Health Care through actions to prevent and treat illnesses, especially in prenatal consultations, is confirmed. The Alyne Network is part of the initiatives to expand care for the mother-child binomial, corresponding to a network with actions focused on pregnancy, child delivery, postpartum and birth. Among its recommendations, it advises that prenatal care should be started early, with risk stratification and referral to other levels of care right from the first consultation.

We conclude that there was an increase in the annual coverage of ultra-processed food consumption and anthropometric data on pregnant women held on the Food and Nutrition Surveillance System. Prevalence of ultra-processed food consumption remained stable and high, with a higher frequency of this food group in the Northeast. Percentage obesity increased in the population studied, but there was no direct association between these factors. Therefore, it is essential to improve the system’s data collection for food and nutritional diagnosis and to incorporate interventions that promote adequate eating practices in this and all stages of life.
