# Supplementary material for: Food and Nutrition Surveillance System: A time series analysis of data coverage, ultra-processed food consumption and obesity among pregnant adults and adolescents, Brazil, 2008-2022
Source: Epidemiol Serv Saude. 2025 Nov 21;34:e20240713. doi: 10.1590/S2237-96222025v34e20240713.en (PMC12642457; doi:10.1590/S2237-96222025v34e20240713.en)
Supplement: Translation

# Translation: Sistema de Vigilância Alimentar e Nutricional: análise de série temporal da cobertura dos dados, consumo de alimentos ultraprocessados e obesidade entre gestantes adultas e adolescentes, Brasil, 2008-2022

**DOI:** 10.1590/S2237-96222025v34e20240713.pt

**Authors:** Sthefani da Costa Penha, Antônio Augusto Ferreira Carioca, Maria Soraia Pinto, Lia Silveira Adriano, Mariana Fialho Bastos, Rosiane de Paes Borges Herculano, Bernard Carl Kendall, Ligia Regina Franco Sansigolo Kerr

**Affiliations:** 1 Universidade Federal do Ceará, Faculdade de Medicina, Programa de Pós-graduação em Saúde Pública, Fortaleza, CE, Brasil; 2 Universidade de Fortaleza, Centro de Ciências da Saúde, Programa de Pós-graduação em Saúde Coletiva, Fortaleza, CE, Brasil; 3 Universidade de Fortaleza, Centro de Ciências da Saúde, Curso de Nutrição, Fortaleza, CE, Brasil; 4 Tulane University, Department of Global Community Health and Behavioral Sciences, New Orleans, LA, Estados Unidos

**Keywords:** Vigilância Alimentar e Nutricional, Saúde Materna, Ingestão de Alimentos, Obesidade, Estudos de Séries Temporais

## Abstract

**Objetivo:**

Avaliar a tendência temporal da cobertura e prevalência do consumo de alimentos ultraprocessados e estado nutricional em gestantes cadastradas no Sistema de Vigilância Alimentar e Nutricional, no âmbito nacional e por macrorregião.

**Métodos:**

Tratou-se de análise de série temporal, utilizando dados de gestantes adultas e adolescentes provenientes dos relatórios públicos de produção e relatórios consolidados do sistema, no âmbito nacional e por macrorregião, de 2008 a 2022. Estimou-se a taxa de variação anual de indicadores da cobertura, consumo alimentar e antropometria por meio da regressão de Prais-Winsten, com nível de significância de 5%.

**Resultados:**

Identificaram-se 319.568 registros de consumo alimentar e 6.585.600 de dados antropométricos. A cobertura anual mostrou tendência crescente de 14,3% para o consumo alimentar (intervalo de confiança de 95% [IC95%] 4,3; 24,7) e de 15,2% para os dados antropométricos (IC95% 10,2; 20,4). O consumo de ultraprocessados apresentou prevalência média de 90,0%, mantendo-se estável e elevada, com maior frequência na região Nordeste. A obesidade evoluiu de 13,3% para 29,9% em gestantes adultas e de 4,5% para 10,4% em gestantes adolescentes, com variação anual de 5,2% e 5,9% (p-valor<0,001).

**Conclusão:**

Houve aumento da cobertura anual do consumo de alimentos ultraprocessados e dos dados antropométricos de gestantes cadastradas no Sistema de Vigilância Alimentar e Nutricional. A prevalência do consumo de ultraprocessados se manteve estável e elevada, com maior frequência desse grupo alimentar no Nordeste. O percentual de obesidade aumentou na população estudada sem, no entanto, evidenciar associação direta entre estes fatores.

**Aspectos éticos**

A presente pesquisa respeitou os princípios éticos, obtendo os seguintes dados de aprovação:

Comitê de ética em pesquisa: Universidade de Fortaleza

Número do parecer: 4348452

Data de aprovação: 16/4/2020

Certificado de apresentação de apreciação ética: 31540320.9.1001.5052

Registro do consentimento livre e esclarecido: Não se aplica.

## Introdução

A gestação produz alterações fisiológicas para adaptar o corpo às suas necessidades e ao desenvolvimento fetal (1). Nesse cenário, o consumo de alimentos ultraprocessados pode repercutir na diminuição da qualidade nutricional, bem como no desenvolvimento de obesidade, diabetes gestacional e pré-eclâmpsia em gestantes, no baixo peso ao nascer e nas alterações no neurodesenvolvimento em crianças (2-5). A inclusão desses alimentos é influenciada por fatores sociodemográficos, entre menor idade materna, baixa escolaridade e multiparidade (6).

A Vigilância Alimentar e Nutricional, terceira diretriz da Política Nacional de Alimentação e Nutrição, faz-se essencial por possibilitar o monitoramento da alimentação e nutrição e os seus determinantes através do Sistema de Vigilância Alimentar e Nutricional (7). Esse sistema foi lançado em 2008 e realiza o registro e a divulgação de informações sobre o consumo alimentar e o estado nutricional da população acompanhada na Atenção Primária do Sistema Único de Saúde (8).

As refeições devem priorizar a inclusão de alimentos in natura ou minimamente processados, com o consumo diário de frutas, legumes e verduras, feijão e leguminosas, e a restrição de ultraprocessados e bebidas com adição de açúcares na rotina alimentar (9). 

Na dieta habitual de gestantes brasileiras, constatou-se a participação de 21,0% de alimentos ultraprocessados (10). Observa-se que, à medida que aumenta a contribuição desses alimentos, há redução da ingestão de alimentos in natura ou minimamente processados. A maior participação de alimentos ultraprocessados se associa à maior ingestão de calorias, carboidratos simples, sódio e gorduras. No entanto, há diminuição do consumo de proteínas, carboidratos complexos, ferro, folato, selênio, zinco e potássio, comprometendo a qualidade alimentar nesse período (11).

Entre grávidas adolescentes, averiguou-se elevada prevalência de excesso de peso no Brasil e nas macrorregiões entre 2008 e 2018. Assim, evidencia-se a modificação no perfil nutricional ao longo dos anos, associando-se ao aumento de intercorrências na gestação e no nascimento (12,13).

A crescente prevalência de doenças relacionadas ao consumo excessivo de alimentos ultraprocessados ​​e à inadequação do estado nutricional, especialmente entre gestantes, ressalta a necessidade do monitoramento contínuo da ingestão alimentar e do estado nutricional. Nesse sentido, tem-se como intuito orientar políticas públicas voltadas à alimentação e à nutrição e aprimorar intervenções nutricionais, contribuindo para a prevenção de condições adversas para mães e filhos. 

Este estudo objetivou avaliar a tendência temporal da cobertura e a prevalência do consumo de alimentos ultraprocessados e estado nutricional e a correlação entre a ingestão de alimentos ultraprocessados e obesidade em gestantes adultas e adolescentes cadastradas no Sistema de Vigilância Alimentar e Nutricional, no âmbito nacional e por macrorregião.

## Métodos

### Delineamento do estudo

Tratou-se de análise de série temporal, utilizando dados secundários disponibilizados pela plataforma do Sistema de Vigilância Alimentar e Nutricional versão Web, referentes ao período 2008-2022. As unidades de análise incluíram o Brasil, as cinco macrorregiões (Norte, Nordeste, Centro-Oeste, Sudeste e Sul), os 26 estados e o Distrito Federal. Os dados foram coletados do sistema em 23 de abril de 2023.

### Contexto

O Sistema de Vigilância Alimentar e Nutricional foi disponibilizado, em sua primeira versão, em 2004 pelo Ministério da Saúde. Em 2008, uma versão para acesso pela internet foi lançada com o objetivo de aprimorar o sistema, abrangendo o registro e a divulgação de informações sobre ingestão alimentar e avaliação nutricional da população acompanhada na Atenção Primária à Saúde. Em 2017, considerando o seu significativo papel, foi lançada a versão 3.0 do sistema, objetivando melhorar a junção do sistema e e-SUS Atenção Básica (8).

O sítio eletrônico compila informações oriundas de ações de vigilância alimentar e nutricional, obtidas a partir dos índices de antropometria e indicadores da ingestão alimentar coletados e registrados por profissionais da saúde, Sistema de Gestão do Programa Bolsa Família, Sistema de Vigilância Alimentar e Nutricional versão Web e e-SUS Atenção Básica (14). O acesso público acontece através de filtros, incluindo mês e ano, região e fase da vida. A partir disso, são gerados dados em planilha eletrônica.

### Participantes

Foram analisados dados da cobertura no Sistema de Vigilância Alimentar e Nutricional de gestantes adultas e adolescentes cadastradas. Para a realização deste estudo, foi considerada a fase da vida da gestante, a qual é caracterizada como qualquer mulher grávida segundo a classificação do sistema (15).

### Variáveis

A taxa de cobertura total de gestantes foi calculada através da divisão do número de registros de consumo alimentar de 2015 a 2022 e estado nutricional de 2008 a 2022 do Sistema de Vigilância Alimentar e Nutricional versão Web pelo número de nascidos vivos desse período e multiplicado por 100, baseando-se em estudo prévio (16). Para o cálculo da estimativa de gestantes, foi utilizado o número de nascidos vivos, coletado do Sistema de Informação sobre Nascidos Vivos por meio de filtros, incluindo ano e região.

A ingestão de alimentos foi avaliada por meio dos indicadores de consumo alimentar. Estes foram analisados através de formulários para obter informações sobre os alimentos consumidos no dia anterior, as quais foram coletadas por profissionais que compõem equipes de Atenção Primária à Saúde. Foram disponibilizados três formulários, sendo um para crianças menores de 6 meses, um para crianças de 6-23 meses e outro para crianças com 2 anos ou mais, adolescentes, gestantes, adultos e idosos. Neste estudo, foi utilizado o formulário para gestantes. O indicador de consumo de alimentos ultraprocessados correspondeu ao de, pelo menos, um marcador de consumo alimentar não saudável no dia anterior, tendo como opções: bebidas adoçadas; macarrão instantâneo, salgadinhos de pacote ou biscoitos salgados; hambúrguer e/ou embutidos; e biscoito recheado, doces ou guloseimas (17).

O estado nutricional foi mensurado pelo índice de massa corporal (IMC) pela semana gestacional e avaliado segundo a classificação proposta por Atalah, parâmetro adotado pelo Sistema de Vigilância Alimentar e Nutricional no Brasil e reconhecido em publicações técnicas do Ministério da Saúde. Essa classificação possibilitou avaliar a evolução nutricional na gestação (18) e compreendeu:

- I – baixo peso (IMC menor ou igual aos valores presentes na coluna do estado nutricional de baixo peso);
- II – adequado (IMC entre os valores presentes na coluna do estado nutricional de baixo peso e adequado); e
- III – sobrepeso ou obesidade (IMC entre os valores presentes na coluna do estado nutricional de sobrepeso ou obesidade.

Os pontos de corte numéricos para a classificação do estado nutricional variaram conforme a semana gestacional, inviabilizando a adoção de valores fixos para cada categoria. Esse índice foi resultante do peso corporal em quilogramas dividido pela estatura em metros ao quadrado. A semana gestacional foi determinada através da data da última menstruação, informação presente e obrigatória no questionário. Esta é de conhecimento da gestante comumente, mas também pode ser coletada por meio de exames realizados na gestação, ou, na ausência de informações confiáveis, a data deve ser estimada com base no último mês de menstruação (15).

### Fonte de dados

Os dados de cobertura foram avaliados por meio dos relatórios públicos de produção. As informações sobre ingestão de alimentos e estado nutricional foram analisadas por meio dos relatórios consolidados por ano no sítio eletrônico do Sistema de Vigilância Alimentar e Nutricional versão Web (https://sisaps.saude.gov.br/sisvan/relatoriopublico/index), com os códigos de análise definidos de acordo com o dado (consumo alimentar ou estado nutricional), ano de referência, mês de referência, região, faixa etária e fase da vida.

### Viés

Com o objetivo de minimizar o risco de vieses, os protocolos do Sistema de Vigilância Alimentar e Nutricional foram aplicados para a exclusão de valores implausíveis. Além disso, há protocolos de padronização dos dados.

### Tamanho do estudo

Foram analisados dados agregados por macrorregiões e Brasil, com 6.585.600 registros para antropometria e 319.568 registros para consumo alimentar de gestantes. O percentual de cobertura do Sistema de Vigilância Alimentar e Nutricional foi calculado pelo número anual de registros dividido pelo número de nascidos vivos desse período e multiplicado por 100. Essa população foi estimada a partir do número de nascidos vivos, coletado do Sistema de Informação sobre Nascidos Vivos, de cada ano, entre 2008 e 2022.

### Métodos estatísticos

Os dados coletados da plataforma do Sistema de Vigilância Alimentar e Nutricional versão Web, para cada macrorregião e ano de referência, foram apresentados em valores relativos (%). A cobertura e a prevalência do consumo de alimentos ultraprocessados e obesidade (variável dependente) foram calculadas segundo a macrorregião e o Brasil para o ano de referência (variáveis independentes).

Para determinar a variação temporal dos indicadores, calcularam-se as prevalências de cada estrato, por ano. Em seguida, foram utilizados modelos de regressão de Prais-Winsten, gerando coeficiente beta (β) e os seus intervalos de confiança de 95%, assumindo o nível de significância de 5%. Esse método é recomendado para estudos ecológicos a fim de minimizar a autocorreção dos resíduos da regressão entre os anos de análise (19). Para a variação anual média da cobertura do consumo de alimentos ultraprocessados e obesidade, foi utilizada a fórmula da taxa de incremento anual (TIA):

TIA=[-1 + (10^β^)] x 100,

na qual β é resultado da regressão de Prais-Winsten e logaritmo de base 10. Tendência de estabilidade foi indicada a partir de p-valor não significante (p-valor≥0,05) e tendência crescente ou decrescente quando p-valor significante (p-valor<0,05), conforme variação anual positiva ou negativa. Essas informações por unidades federativas foram utilizadas para a correlação de Pearson entre as taxas de incremento anual da ingestão de alimentos ultraprocessados e obesidade entre 2015 e 2022. Utilizou-se o programa Stata versão 11.2 (Stata Corp, College Station, Texas, Estados Unidos) para a realização de todas as análises estatísticas.

## Resultados

Entre 2008 e 2022, foram identificados 6.585.600 registros de antropometria e 319.568 de consumo alimentar de grávidas com cadastro no Sistema de Vigilância Alimentar e Nutricional. De 2015 a 2022, houve aumento da cobertura dos dados de indicadores do consumo de alimentos ultraprocessados de 0,7% a 2,3% (TIA 14,3%; IC95% 4,3; 24,7). Do total de registros dos dados de indicadores do consumo alimentar, 49,4% eram do Sudeste, 19,5% do Nordeste e 12,3% do Sul. A cobertura dos dados antropométricos passou de 3,9% em 2008 para 34,1% em 2022, com variação anual significante de 15,2% (IC95% 10,2; 20,4) (Tabela 1). A maioria dos registros de dados antropométricos foi apresentada pelo Nordeste (35,0%), seguida pelas regiões Sudeste (32,3%) e Norte (11,6%).

**Tabela 1:** Taxa de incremento anual (TIA) e intervalo de confiança de 95% (IC95%) da cobertura do indicador de consumo de alimentos ultraprocessados (%) e dados antropométricos (%) de gestantes cadastradas no Sistema de Vigilância Alimentar e Nutricional. Brasil, 2015-2022 (n=8.872.011)

Ano	n	Consumo de alimentos ultraprocessados (n)	Consumo de alimentos ultraprocessados (%)	Cobertura do indicador de consumo de alimentos ultraprocessados (%)
2015	21.163	16.753	82,0	0,7
2016	32.308	31.137	81,0	1,1
2017	42.120	33.144	81,0	1,4
2018	42.993	32.270	76,0	1,5
2019	38.561	29.243	77,0	1,4
2020	33.561	25.059	76,0	1,2
2021	49.046	39.506	82,0	1,9
2022	59.816	45.382	77,0	2,3
TIA (IC95%)				14,1 (4,3; 24,7)
	n	Obesidade (n)	Obesidade (%)	Cobertura de dados antropométricos (%)
2008	115.406	115.406	11.2	3,9
2009	209.099	209.099	12,6	7,3
2010	252.620	252.620	13,1	8,8
2011	318.150	318.150	14,4	10,9
2012	405.533	405.533	15,2	14,0
2013	483.965	483.965	15,5	16,7
2014	501.638	501.638	16,3	16,8
2015	692.975	692.975	16,9	23,0
2016	848.533	848.533	17,2	29,7
2017	733.089	733.089	18,2	25,1
2018	728.695	728.695	19,6	24,7
2019	629.527	629.506	20,4	22,1
2020	1.031.416	1.031.380	23,1	37,8
2021	1.047.717	1.047.711	23,9	39,1
2022	873.648	873.586	24,4	34,1
TIA (IC95%)				15,2 (10,2; 20,4)

A variação percentual anual do consumo de ultraprocessados se manteve estável. No entanto, houve a participação média de 90,0% destes na dieta de gestantes brasileiras. No Sul, o percentual de grávidas adultas registradas que consumiram hambúrgueres e/ou embutidos cresceu, em média, 7,9% ao ano (p-valor 0,005), sendo observados o aumento de 3,6% por ano do consumo de macarrão instantâneo, salgadinho de pacote ou biscoito salgado (p-valor 0,006) e a elevação de 3,3% do consumo de biscoito recheado, doces ou guloseimas (p-valor 0,003). O consumo de bebidas adoçadas aumentou 1,5% por ano no Nordeste, com redução de 1,9% no Norte (p-valor 0,020) (Tabelas 2 e 3).

No Nordeste, o percentual de gestantes adultas que consumiram macarrão instantâneo, salgadinho de pacote ou biscoito salgado no dia anterior cresceu, em média, 1,8% ao ano (p-valor<0,001). Da mesma forma, foi observado o aumento de 1,6% por ano do consumo de biscoito recheado, doces ou guloseimas (p-valor 0,002). No Norte, ocorreu a diminuição de 3,8% ao ano do consumo de macarrão instantâneo, salgadinho de pacote ou biscoito salgado (p-valor 0,037) e de 2,6% do consumo de biscoito recheado, doces ou guloseimas (p-valor 0,034) (Tabela 2).

**Tabela 2:** Taxa de incremento anual (TIA) e p-valor do indicador de consumo de alimentos ultraprocessados (%) em gestantes adultas cadastradas no Sistema de Vigilância Alimentar e Nutricional. Brasil e macrorregiões, 2015-2022 (n=319.715)

Ano	Centro-Oeste (%)	Nordeste (%)	Norte (%)	Sudeste (%)	Sul (%)	Brasil (%)
2015	85,0	72,0	80,0	82,0	84,0	81,0
2016	82,0	70,0	77,0	82,0	83,0	80,0
2017	81,0	72,0	75,0	82,0	82,0	79,0
2018	76,0	71,0	78,0	74,0	82,0	75,0
2019	79,0	73,0	73,0	76,0	83,0	76,0
2020	81,0	71,0	74,0	76,0	79,0	75,0
2021	87,0	76,0	77,0	82,0	88,0	81,0
2022	95,0	92,0	90,0	92,0	93,0	92,0
TIA (p-valor)	1,5 (0,324)	2,7 (0,074)	1,0 (0,426)	1,2 (0,443)	1,2 (0,214)	1,5 (0,345)
**Consumo de hambúrgueres e/ou embutidos**						
2015	43,0	33,0	44,0	35,0	36,0	36,0
2016	33,0	32,0	35,0	32,0	37,0	33,0
2017	34,0	33,0	31,0	32,0	37,0	33,0
2018	32,0	33,0	34,0	29,0	35,0	31,0
2019	37,0	32,0	26,0	36,0	43,0	35,0
2020	47,0	34,0	26,0	37,0	44,0	36,0
2021	61,0	46,0	39,0	49,0	66,0	50,0
2022	41,0	37,0	34,0	41,0	51,0	41,0
TIA (p-valor)	4,5 (0,210)	3,5 (0,040)	-2,5 (0,436)	4,7 (0,057)	7,9 (0,005)	4,1 (0,078)
**Consumo de bebidas adoçadas**						
2015	65,0	49,0	54,0	64,0	65,0	61,0
2016	63,0	47,0	59,0	62,0	62,0	60,0
2017	60,0	46,0	54,0	62,0	60,0	58,0
2018	58,0	47,0	57,0	57,0	60,0	55,0
2019	57,0	50,0	53,0	58,0	63,0	56,0
2020	61,0	49,0	51,0	57,0	59,0	55,0
2021	72,0	54,0	55,0	63,0	70,0	62,0
2022	61,0	50,0	52,0	60,0	64,0	57,0
TIA (p-valor)	0,2 (0,874)	1,5 (0,034)	-1,9 (0,020)	-0,7 (0,371)	0,7 (0,345)	-0,6 (0,499)
**Consumo de macarrão instantâneo, salgadinho de pacote ou biscoito salgado**						
2015	43,0	33,0	44,0	35,0	36,0	36,0
2016	33,0	32,0	35,0	32,0	37,0	33,0
2017	34,0	33,0	31,0	32,0	37,0	33,0
2018	32,0	33,0	34,0	29,0	35,0	31,0
2019	34,0	35,0	30,0	30,0	40,0	33,0
2020	38,0	35,0	30,0	32,0	39,0	34,0
2021	42,0	36,0	31,0	34,0	46,0	36,0
2022	36,0	36,0	31,0	34,0	45,0	36,0
TIA (p-valor)	0,2 (0,905)	1,8 (<0,001)	-3,8 (0,037)	-0,1 (0,938)	3,6 (0,006)	0,5 (0,677)
**Consumo de biscoito recheado, doces ou guloseimas**						
2015	49,0	32,0	43,0	45,0	42,0	43,0
2016	42,0	34,0	40,0	44,0	43,0	42,0
2017	43,0	37,0	39,0	45,0	43,0	43,0
2018	40,0	36,0	38,0	39,0	42,0	39,0
2019	40,0	37,0	35,0	40,0	47,0	40,0
2020	39,0	35,0	33,0	40,0	43,0	38,0
2021	53,0	39,0	37,0	44,0	55,0	44,0
2022	42,0	36,0	36,0	42,0	50,0	41,0
TIA (p-valor)	0,0 (0,992)	1,6 (0,026)	-2,6 (0,034)	-1,0 (0,353)	3,3 (0,003)	-0,5 (0,517)

No grupo de gestantes adolescentes, a variação percentual do consumo de bebidas adoçadas no dia anterior diminuiu 1,0% entre 2015 e 2022 no Brasil (p-valor 0,034) e 1,6% no Norte (p-valor 0,027). Contudo, nesses sete anos, o número de grávidas adolescentes que consumiram hambúrgueres e/ou embutidos cresceu 4,6% ao ano no Nordeste (p-valor 0,005). No Sul, verificou-se o aumento de 3,5% por ano do consumo de hambúrgueres e/ou embutidos (p-valor 0,003) e de 1,5% do consumo de biscoito recheado, doces ou guloseimas (p-valor 0,002) (Tabela 3). 

**Tabela 3:** Taxa de incremento anual (TIA) e p-valor do indicador de consumo de alimentos ultraprocessados (%) em gestantes adolescentes cadastradas no Sistema de Vigilância Alimentar e Nutricional. Brasil e macrorregiões, 2015-2022 (n=42.911)

Ano	Centro-Oeste (%)	Nordeste (%)	Norte (%)	Sudeste (%)	Sul (%)	Brasil (%)
2015	90,0	80,0	84,0	90,0	93,0	87,0
2016	88,0	82,0	83,0	89,0	88,0	87,0
2017	85,0	83,0	79,0	88,0	90,0	86,0
2018	80,0	77,0	83,0	83,0	89,0	82,0
2019	88,0	79,0	77,0	83,0	88,0	81,0
2020	87,0	79,0	79,0	84,0	86,0	82,0
2021	90,0	81,0	83,0	87,0	90,0	85,0
2022	95,0	90,0	89,0	91,0	91,0	91,0
TIA (p-valor)	0,7 (0,415)	0,9 (0,293)	0,5 (0,596)	0,0 (0,959)	-0,2 (0,568)	0,5 (0,633)
**Consumo de hambúrgueres e/ou embutidos**						
2015	47,0	32,0	38,0	41,0	46,0	40,0
2016	39,0	37,0	31,0	42,0	43,0	39,0
2017	39,0	37,0	40,0	41,0	47,0	39,0
2018	35,0	34,0	33,0	39,0	50,0	38,0
2019	33,0	39,0	30,0	41,0	50,0	39,0
2020	51,0	44,0	30,0	45,0	46,0	42,0
2021	63,0	47,0	42,0	49,0	58,0	48,0
2022	49,0	42,0	38,0	44,0	56,0	44,0
TIA (p-valor)	3,3 (0,409)	4,6 (0,005)	0,7 (0,733)	1,8 (0,130)	3,5 (0,003)	2,2 (0,083)
**Consumo de bebidas adoçadas**						
2015	69,0	53,0	61,0	71,0	75,0	67,0
2016	67,0	58,0	64,0	70,0	69,0	67,0
2017	69,0	57,0	59,0	70,0	71,0	66,0
2018	62,0	53,0	64,0	65,0	69,0	63,0
2019	66,0	56,0	57,0	66,0	71,0	63,0
2020	71,0	55,0	54,0	67,0	68,0	62,0
2021	70,0	57,0	59,0	67,0	69,0	64,0
2022	74,0	56,0	57,0	67,0	71,0	63,0
TIA (p-valor)	1,0 (0,256)	0,2 (0,673)	-1,6 (0,027)	-0,9 (0,094)	-0,5 (0,115)	-1,0 (0,034)
**Consumo de macarrão instantâneo, salgadinho de pacote ou biscoito salgado**						
2015	48,0	43,0	49,0	47,0	50,0	47,0
2016	43,0	44,0	41,0	43,0	47,0	43,0
2017	45,0	42,0	38,0	41,0	47,0	42,0
2018	39,0	39,0	43,0	38,0	47,0	40,0
2019	44,0	42,0	37,0	38,0	51,0	40,0
2020	45,0	43,0	36,0	41,0	48,0	41,0
2021	51,0	43,0	41,0	43,0	49,0	44,0
2022	51,0	42,0	43,0	41,0	53,0	43,0
TIA (p-valor)	1,6 (0,325)	-0,2 (0,788)	-1,4 (0,382)	-1,6 (0,291)	0,9 (0,117)	-1,0 (0,404)
**Consumo de biscoito recheado, doces ou guloseimas**						
2015	58,0	46,0	50,0	57,0	55,0	54,0
2016	52,0	49,0	50,0	56,0	54,0	53,0
2017	53,0	50,0	46,0	55,0	58,0	53,0
2018	48,0	45,0	45,0	51,0	55,0	50,0
2019	48,0	47,0	43,0	48,0	59,0	48,0
2020	54,0	46,0	41,0	51,0	54,0	49,0
2021	60,0	48,0	46,0	54,0	63,0	52,0
2022	56,0	45,0	45,0	51,0	59,0	50,0
TIA (p-valor)	0,3 (0,869)	-0,6 (0,209)	-1,7 (0,130)	-1,5 (0,152)	1,5 (0,002)	-1,1 (0,149)

Entre 2008 e 2022, a prevalência de obesidade em gestantes adultas passou de 13,3% para 29,9%, com o aumento de 5,2% ao ano em todas as macrorregiões e no Brasil (p-valor<0,001) (Tabela 4). No grupo de gestantes adolescentes, houve o aumento anual de 5,9% de obesidade, evoluindo de 4,5% em 2008 para 10,4% em 2022 (p-valor<0,001) (Tabela 5).

**Tabela 4:** Taxa de incremento anual (TIA) e p-valor da prevalência (%) da obesidade em gestantes adultas cadastradas no Sistema de Vigilância Alimentar e Nutricional. Brasil e macrorregiões, 2008-2022 (n=1.566.144)

Ano	Centro-Oeste (%)	Nordeste (%)	Norte (%)	Sudeste (%)	Sul (%)	Brasil (%)
2008	14,1	9,8	9,4	15,6	17,1	13,3
2009	14,5	11,0	8,9	16,7	18,3	14,9
2010	15,2	11,5	8,9	18,1	18,4	15,5
2011	17,4	13,1	10,6	19,6	19,8	16,9
2012	18,1	14,9	11,2	20,1	21,6	17,7
2013	19,0	14,5	11,9	20,0	22,2	18,1
2014	20,2	15,7	13,2	20,7	22,9	19,0
2015	23,1	16,8	14,7	21,8	24,0	19,6
2016	23,8	17,6	15,0	22,8	24,5	20,2
2017	22,2	18,7	15,9	24,3	26,1	21,3
2018	23,6	20,4	17,3	25,6	27,6	22,5
2019	24,4	21,2	18,2	26,1	27,8	23,2
2020	25,5	23,6	20,5	29,4	29,3	26,1
2021	25,7	24,2	21,0	29,6	29,7	26,6
2022	26,0	24,4	21,6	29,8	29,8	29,9
TIA (p-valor)	4,6 (<0,001)	6,8 (<0,001)	7,1 (<0,001)	4,7 (<0,001)	4,0 (<0,001)	5,2 (<0,001)

**Tabela 5:** Taxa de incremento anual (TIA) e p-valor da prevalência (%) da obesidade em gestantes adolescentes cadastradas no Sistema de Vigilância Alimentar e Nutricional. Brasil e macrorregiões, 2008-2022 (n=139.144)

Ano	Centro-Oeste (%)	Nordeste (%)	Norte (%)	Sudeste (%)	Sul (%)	Brasil (%)
2008	4,3	3,4	2,8	4,6	6,4	4,5
2009	5,2	3,0	2,2	5,5	7,1	5,0
2010	4,8	3,6	2,4	6,1	6,9	5,2
2011	6,3	4,1	2,8	7,2	7,9	6,0
2012	6,7	4,5	3,0	6,9	8,5	6,2
2013	6,8	4,5	3,3	7,0	8,9	6,3
2014	6,7	4,9	3,6	7,4	9,3	6,6
2015	10,5	5,9	4,8	8,0	9,9	7,1
2016	9,9	6,2	4,7	8,0	10,0	7,1
2017	7,8	6,4	4,8	8,8	10,4	7,3
2018	8,1	7,2	5,3	9,6	11,2	7,9
2019	8,5	7,7	6,0	10,2	11,6	8,5
2020	9,5	8,8	6,9	11,5	13,2	9,7
2021	10,3	9,3	7,2	12,4	14,3	10,4
2022	10,6	9,4	6,9	12,1	13,9	10,4
TIA (p-valor)	5,9 (<0,001)	8,6 (<0,001)	9,1 (<0,001)	6,7 (<0,001)	5,7 (<0,001)	5,9 (<0,001)

As taxas de incremento anuais das unidades federativas referentes ao consumo de ultraprocessados e obesidade de gestantes adultas não apresentaram correlação significativa (r=0,19; p-valor 0,382). Concomitante, não foi observada correlação significante entre tais variáveis em gestantes adolescentes (r=-0,07; p-valor 0,763).

## Discussão

Este estudo observou aumento da cobertura dos indicadores de consumo alimentar, com a maioria dos registros apresentados pelo Sudeste, e dos dados antropométricos, com a maior frequência de dados pelo Nordeste. Verificou-se estabilidade da ingestão de alimentos ultraprocessados pelas gestantes no Brasil, porém foi observada maior frequência desse grupo alimentar no Nordeste, maior inclusão de hambúrgueres e/ou embutidos na alimentação de gestantes adultas no Sul e diminuição do consumo de bebidas adoçadas por gestantes no Brasil e no Norte. Entre 2008 e 2022, houve aumento da prevalência de obesidade na população estudada, podendo refletir a ingestão contínua de ultraprocessados no Brasil, alimentos constituídos pelo excesso de calorias e baixa qualidade nutricional. Estes achados reforçaram a importância da Vigilância Alimentar e Nutricional como estratégia fundamental para monitorar os determinantes da saúde materno-infantil e orientar ações no âmbito do sistema de saúde.

Os resultados apresentados possuem limitações que devem ser consideradas ao serem interpretados. Por se tratar de análise de série temporal, não é possível extrapolar as suas conclusões agregadas no nível individual. A utilização de dados secundários está inerente à incompletude e inconsistência das informações. No entanto, o Sistema de Vigilância Alimentar e Nutricional corresponde à significativa fonte de dados para a realização de vigilância alimentar e nutricional, pois tem sido aprimorado como ferramenta de cobertura e garantia da qualidade das informações, tornando-se essencial para a gestão e a criação de políticas públicas. Este estudo contou com a utilização de formulários que avaliaram o consumo alimentar do dia anterior através de questões objetivas, minimizando os vieses de memória.

O aumento da cobertura dos indicadores de consumo de ultraprocessados e dados antropométricos em gestantes analisados sugeriu melhor monitoramento nutricional. Apesar da tendência crescente de cobertura, esses percentuais estavam abaixo do esperado ao serem considerados representativos dessa população no nível nacional. No entanto, a literatura indicou evolução na cobertura após a inclusão dos dados pelo e-SUS Atenção Básica ao sistema (8,13). 

Entre os usuários cadastrados no Sistema de Vigilância Alimentar e Nutricional em 2008 e 2017, houve maior variação anual no aumento da cobertura do grupo de mulheres grávidas (19). Tais resultados podem ser explicados pelo maior acompanhamento dos dados coletados desse público durante a assistência pré-natal pela Estratégia Saúde da Família. A avaliação do estado nutricional está entre os condicionantes de saúde consolidados para acesso ao Programa Bolsa Família (13), responsável pela transferência de renda, a fim de amenizar as desigualdades sociais.

A maioria dos registros dos indicadores de consumo alimentar foi proveniente do Sudeste, enquanto os dados antropométricos foram mais frequentemente registrados no Nordeste. Esse padrão também foi identificado em estudo ecológico sobre a cobertura do Sistema de Vigilância Alimentar e Nutricional e o estado nutricional da população idosa no Brasil, que mostrou as maiores variações na disponibilidade dos dados nessas duas regiões (20). Tais resultados podem estar relacionados às diferenças socioeconômicas entre essas regiões: o Sudeste, por contar com maior infraestrutura e recursos, pode absorver melhor os avanços do sistema; e o Nordeste, por concentrar maior número de beneficiários do Programa Bolsa Família, pode apresentar priorização na coleta dos dados antropométricos para o monitoramento do estado nutricional da população.

Embora tenha sido averiguada estabilidade do consumo de alimentos ultraprocessados por gestantes no Brasil, a prevalência de 90,0% indicou ingestão elevada desse grupo alimentar, estando acima do consumo médio de 18,0% da população brasileira (21). Ao comparar as regiões brasileiras de 2015 a 2022, identificou-se a maior frequência do consumo de ultraprocessados pelo Nordeste. Nas regiões Sul e Nordeste, observou-se a maior participação de hambúrgueres e/ou embutidos e, no Norte, a menor ingestão de macarrão instantâneo, salgadinho de pacote ou biscoito salgado por gestantes adultas. Consistente com esses resultados, observou-se que mulheres italianas em gestação gemelar apresentaram consumo excessivo de carnes e peixes processados (22). O elevado consumo de ultraprocessados e a sua manutenção ao longo do tempo são característicos do sistema alimentar hegemônico, no qual há domínio na estrutura de produção, distribuição e consumo de alimentos mundialmente como resultado da industrialização e globalização (23). 

Somando-se a isso, a pandemia de covid-19 modificou os hábitos alimentares, estimulando a maior inclusão de ultraprocessados em detrimento dos alimentos in natura e minimamente processados, sendo constatado pelo aumento dos percentuais em 2020, ano em que iniciou o período pandêmico. Nessa época, foi constatado aumento do armazenamento de alimentos em adultos com obesidade, apontando-se como resultado das dificuldades econômicas e do isolamento social vivenciados. Nessa categoria, destacaram-se os alimentos com maior densidade energética, menor qualidade nutricional e maior prazo de validade, como os ultraprocessados (24).

A redução no consumo de bebidas adoçadas por gestantes no Brasil e na região Norte coincide com o encontrado pela Pesquisa de Orçamentos Familiares de 2017 e 2018 (25) e nos dados da Vigilância de Fatores de Risco e Proteção para Doenças Crônicas por Inquérito Telefônico de 2013 e 2023 (21,25), que constataram o decréscimo da participação de refrigerantes nos domicílios brasileiros nos últimos anos. Sugere-se que o planejamento e o aperfeiçoamento de políticas públicas, como a publicação do Guia Alimentar para a População Brasileira em sua segunda edição em 2014 e de seus fascículos, são essenciais para essas mudanças.

Constatou-se tendência crescente de obesidade em gestantes adultas e adolescentes no Brasil e por todas as macrorregiões, reforçando os achados do estudo ecológico com gestantes adolescentes participantes do Programa Bolsa Família, que constatou elevação das prevalências de sobrepeso e obesidade entre 2008 e 2018, com variação anual de 8,0% (12). O aumento da prevalência de obesidade para gestantes no país é característico da chamada transição nutricional, já observada na população adulta e intensificada pela pandemia de covid-19 (27). A evolução do ganho de peso gestacional é influenciada pelo estado nutricional prévio à gravidez, como foi observado na evolução ponderal de gestantes portuguesas (28). 

Condições socioeconômicas, a exemplo de escolaridade e renda, são apontadas como os principais fatores que exercem efeitos progressivos do ambiente pré-gestacional sobre o ganho de peso na gestação (29). Somando-se a isso, está o atual cenário de sindemia global, que é definida como um conjunto de pandemias que acometem a população em um mesmo local e período, entre obesidade, desnutrição, alterações climáticas e insegurança alimentar e nutricional (caracterizada pelo consumo de alimentos calóricos e escassos em nutrientes essenciais) (30).

Nesse contexto, confirma-se a relevância de aprimorar o acesso ao cuidado adequado na Atenção Primária à Saúde através de ações de prevenção e tratamento de enfermidades, sobretudo nas consultas de pré-natal. A Rede Alyne está inserida entre as iniciativas de ampliação do cuidado ao binômio materno-infantil, correspondendo a uma rede com ações voltadas a gestação, parto, puerpério e nascimento. Entre as suas recomendações, orienta que o pré-natal seja iniciado precocemente, com estratificação de risco e encaminhamento a outros níveis de atenção desde a primeira consulta.

Conclui-se que houve aumento da cobertura anual do consumo de alimentos ultraprocessados e dados antropométricos de gestantes no Sistema de Vigilância Alimentar e Nutricional. A prevalência do consumo de ultraprocessados se manteve estável e elevada, com maior frequência desse grupo alimentar no Nordeste. O percentual de obesidade aumentou na população estudada sem, no entanto, evidenciar associação direta entre estes fatores. Diante disso, tornam-se essenciais o aprimoramento da coleta de dados do sistema para o diagnóstico alimentar e nutricional e a incorporação de intervenções que promovam práticas alimentares adequadas nessa e em todas as fases da vida.
